# Mucocele of the Paranasal Sinuses: Retrospective Analysis of a Series of Eight Cases

**DOI:** 10.7759/cureus.41986

**Published:** 2023-07-17

**Authors:** Nasir A Magboul, Atheer A Alzubaidi, Lamya A Abumsmar, Ali Alzarei, Mohammad Al-Ahmari, Mona A Alshehri

**Affiliations:** 1 ENT, Armed Forces Hospital, Jazan, SAU; 2 Otolaryngology - Head and Neck Surgery, Khamis Mushait General Hospital, Khamis Mushait, SAU; 3 Otolaryngology - Head and Neck Surgery, Aseer Central Hospital, Abha, SAU; 4 Otolaryngology, Aseer Central Hospital, Abha, SAU

**Keywords:** imaging study, saudi arabia, endoscopic approach, para-nasal sinus, mucocele

## Abstract

Background: Paranasal sinus mucoceles are epithelium-lined cystic masses usually resulting from obstruction of sinus ostia. They most frequently occur in the frontal and ethmoid sinuses. The etiology is not clarified, but the most common identifiable cause of mucoceles following functional endoscopic sinus surgery (FESS), trauma, neoplasms, and allergy. The clinical symptoms of mucocele vary and are not specific, the most common being ophthalmic symptoms and headache, impinging on adjacent orbital structures, and causing ophthalmic sequelae such as double vision, commonly followed by orbital swelling, epiphora, proptosis, and ptosis. All patients in this study had frontal and frontoethmoidal mucocele and initially complained of frontal headache and ophthalmic symptoms. Definitive treatment options for paranasal sinus mucoceles include external approaches and endoscopic marsupialization.

Objective: The study aimed to identify the etiology, clinical presentation, most common para nasal sinus affected by mucocele, management, and the rate of recurrence in eight cases with mucocele of the paranasal sinuses.

Methods: Eight patients diagnosed with mucocele of the paranasal sinuses were admitted to our institution between 2014 and 2021. There were two females and six males aged between 14 and 67. Initial symptoms, duration, clinical presentation upon admission, location of the mucocele, type of surgical intervention, and outcome have all been studied.

Results: The most common symptoms at diagnosis were orbital involvement, retrobulbar, and frontal headache. Most patients were diagnosed with frontal mucocele (40%), and three were frontoethmoidal mucocele at the time of presentation. The rest of the cases were diagnosed with ethmoidal mucocele (25%). The etiology was identified in four patients and was unclear in the rest. All patients underwent endoscopic sinus surgery. The most identifiable postoperative complication was a headache.

Conclusions: The endonasal endoscopic approach is a safe and effective treatment for paranasal sinus mucocele and provides adequate drainage with a low recurrent rate.

## Introduction

The obstruction of the sinus ostia causes epithelium-lined cystic tumors known as paranasal sinus mucoceles. Most commonly, the frontal and ethmoid sinuses are where mucoceles develop. The disease etiology is not entirely clarified, but some studies have observed that functional endoscopic sinus surgery (FESS) is the most commonly identifiable cause [1-3]. Paranasal sinus mucoceles are benign cystic lesions [4]. Primary mucoceles occur in patients with no previous sinus surgery history or known cause of mucoceles, and secondary mucoceles result as a complication of endoscopic sinus surgery or the Caldwell-Luc operation [5]. This is in line with studies linking the pathology of mucocele with inflammatory mediators [6]. The differential diagnoses include dermoid and epidermoid cysts, angiofibroma, neurofibroma, osseous fibroma, cholesterol granuloma, and odontogenic cysts. Many of these lesions cause expansion similar to that caused by mucoceles in the sinus wall [7].

Mucoceles may erode into the intraorbital or intracranial spaces [2]. Ophthalmologic symptoms most commonly occur because of the impingement on adjacent orbital structures and can cause ophthalmic sequelae, such as decreased visual acuity. A study found that 44.9% of mucoceles had an intraorbital extension, intracranial extension, or both [2]. Patients have also reported rhinological or neurological complaints [1,4]. It is characterized by resorption of the surrounding osseous walls and results in significant facial deformity [8]. The clinical symptoms vary, but the most common symptom reported so far is headache followed by maxillofacial pressure and nasal congestion [2]. Bouatay et al. reported that computed tomography (CT) scans enable the objectification of intraorbital extensions [8]. The CT scan is the modality of choice to diagnose mucocele as it provides essential anatomical details, highlights how it interacts with nearby bony structures, and helps with surgical designs [1,8]. The definitive treatment for these patients is surgery. Endoscopic procedures and mucocele marsupialization are carried out with extreme care taken to protect the mucosa of the natural sinus drainage route [2].

## Materials and methods

Study design, setting, and date

This retrospective, hospital file-based study was conducted at the Asser Central Hospital, Southern Region, Saudi Arabia, between January and April, 2022. The hospital records of patients who underwent endoscopic sinus surgery for marsupialization of paranasal mucocele during 2014-2021 were reviewed. Eight patients diagnosed with mucocele of the paranasal sinuses were admitted to our institution between 2014 and 2021. A data collection sheet was employed to record the extracted data, which included the patient’s age, sex, clinical presentation upon admission, location of the mucocele, type of surgical intervention, and outcome.

Ethical considerations

The study protocol was approved by the Ethics Committee of the Asser Central Hospital, Asser Institutional Review Board, Abha, Saudi Arabia (approval number: REC-09-05-2022). Confidentiality of the participants’ data was ensured by keeping the data collection sheets anonymous after assigning a code number to each patient.

Data analysis

The data were extracted, revised, coded, and processed using IBM SPSS Statistics for Windows, Version 22.0 (Released 2013; IBM Corp., Armonk, New York, United States).

## Results

**Figure 1:**
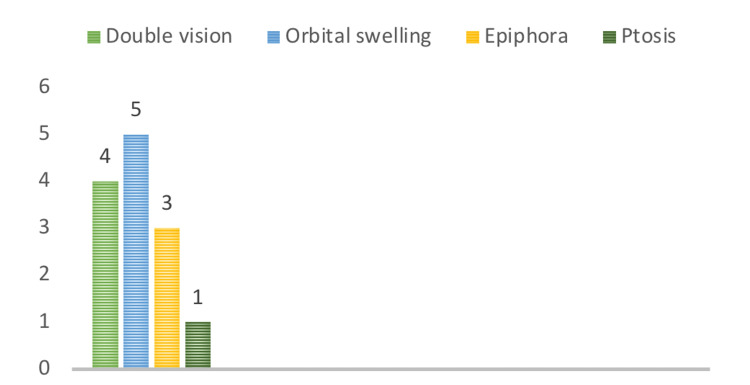
Eye symptoms

**Figure 2:**
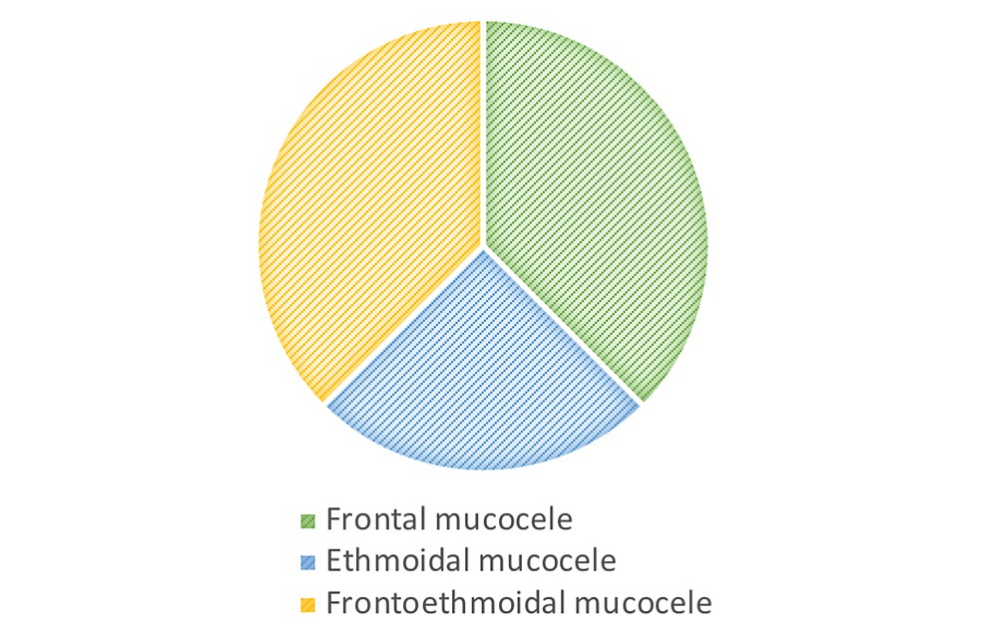
Localization of mucocele

**Figure 3:**
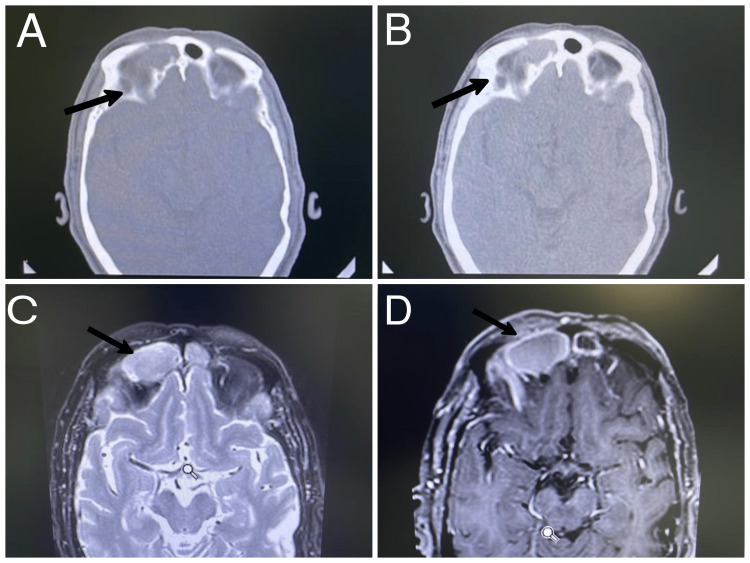
Case 1. Right frontal mucocele in male patient presenting with right eye ptosis, diplopia, and headache. (A) Axial CT scan bone window at level of the frontal sinus, showing expansile mass in right frontal sinus with bone thinning in both anterior and posterior table; (B) Soft tissue window; (C) T1 weighted MRI with gadolinium axial cut showing low signal with peripheral enhancement intensity; (D) T2 FLAIR MRI indicating high signal intensity lesion. FLAIR: fluid-attenuated inversion recovery

**Figure 4:**
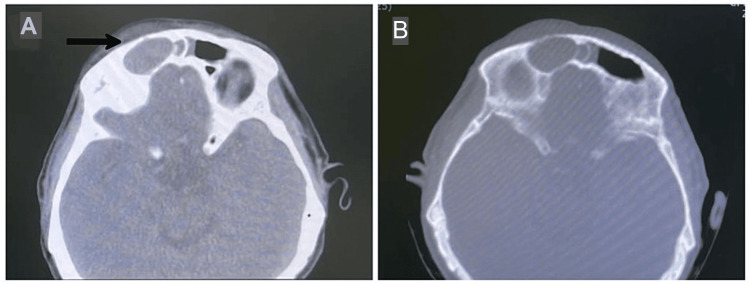
Case 3. Axial CT scan bone window at level of fronto-ethmoidal area showing expansile lesion occupying the right ethmoid (arrow), and right frontal area with intact anterior and posterior table of frontal bone.

**Figure 5:**
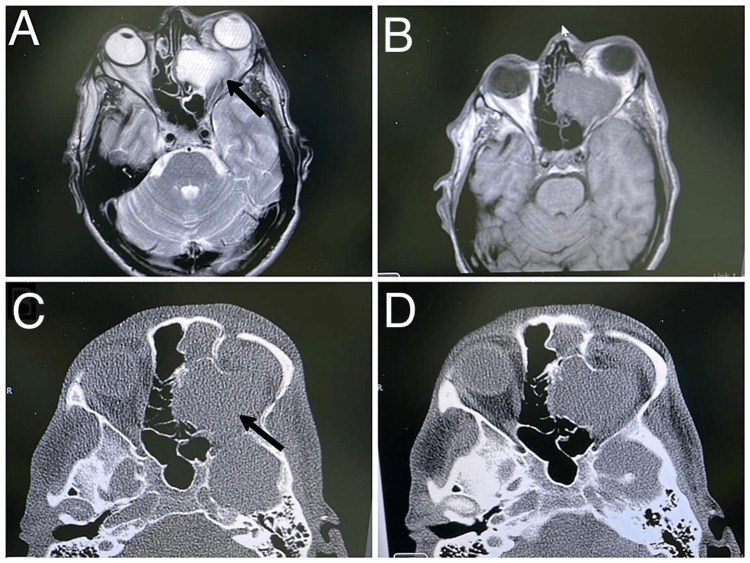
Case 6. (A,B) Left fronto-ethmoidal mucocele with obstructed left frontal drainage and associated with left unilateral proptosis (low signal intensity in T1 and high signal in T2); it is significantly encroaching upon the left orbital cavity, displacing and compressing left orbital structures without gross invasion; (C,D) CT scan showing left fronto-ethmoidal lobulated cystic lesion measures about 5 x 4 x 2.8 cm, with extending mainly to left retrobulbar space with associated left proptosis; there is associated expansion/remodelling and erosions of the left lamina papyracea and medial superior orbital wall.

**Figure 6:**
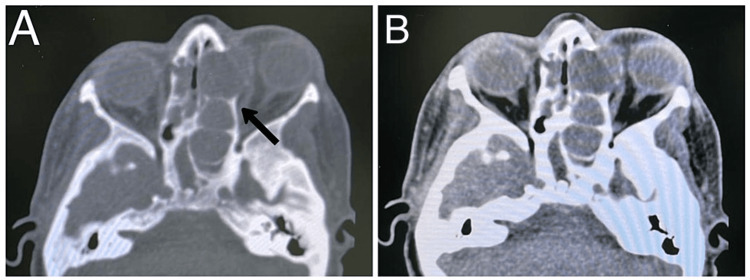
Case 7. Fronto-ethmoidal mucocele with proptosis, diplopia, and headache. Axial CT scan bone window at level of fronto-ethmoidal area showing expansile lesion occupying of the left anterior ethmoid (arrow), and left frontal with lateral displacement of lamina papyracea.

**Figure 7:**
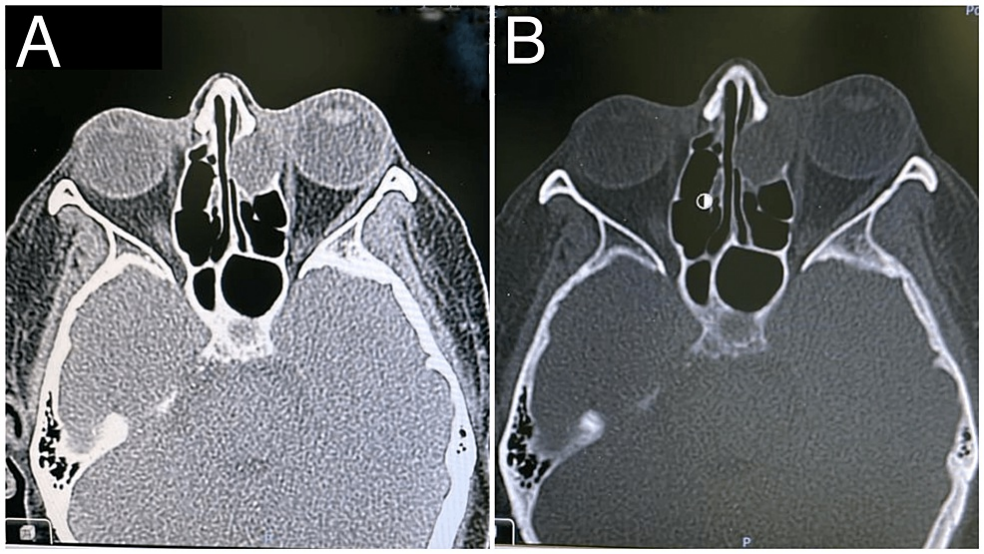
Case 8. Left ethmoiddal mucocele presenting with left nasal duct obstruction, epiphora, and left side facial headache.

A total of eight patients were admitted as mucocele cases between 2014 and 2021. There were six males (75%) and two females (25%). Clinical symptoms, their duration, and suggested etiology have been summarized in Table 1. The most common symptoms at the time of diagnosis were related to the orbit. The most common group of symptoms identified in all patients were double vision in four patients, orbital swelling in five patients, epiphora in three patients, and ptosis in one patient (Figure 1). Retrobulbar and frontal headaches were observed in four patients (50%). The majority of patients were diagnosed with frontal mucocele (40%), and three patients had frontoethmoidal mucocele at the time of presentation (Figure 2). The rest of the patients were diagnosed with ethmoidal mucocele (25%). The etiology was identified in four patients and was unclear in the rest. All patients underwent endoscopic sinus surgery. Headache was the most identifiable postoperative symptom, which was reported in five patients. Figures 3-7 show the CT images of cases 1, 3, 5, 7, and 8.

**Table 1:** Clinical presentation and siggested etiology of the patients F: female; M: male; FESS: functional endoscopic sinus surgery

Patient number	Gender/ age	Suggested etiology	Localization of mucocele	Clinical presentation and duration
1	M/67	FESS	Right frontal	Right eye ptosis, double vision, frontal headache, and epiphora for three years.
2	M/52	History of old nasal trauma, mucocele evacuation by FESS done twice	Left ethmoidal	Left eye proptosis, and epiphora for 10 years.
3	M/55	Unknown	Right frontal and ethmoidal	Right supraorbital swelling, pain, and headache for six days.
4	M/14	Unknown	Left frontal	Unknown.
5	M/45	History of same presentation and operated twice externally	Left frontal	Left nasal obstruction, left orbital swelling, and double vision for one year.
6	M/35	Unknown	Left frontal and ethmoidal	Left eye orbital swelling, double vision, poor smell sensation, nasal obstruction for two months.
7	F/39	History of nasal polyposis and four times FESS	Left frontal and ethmoidal	Left eye proptosis, double vision, frontal headache for two months.
8	F/44	Unknown	Left ethmoid	Left nasal duct obstruction, epiphora, left side nasal obstruction, left side facial headache for one year.

## Discussion

Epithelium-lined, mucus-filled cystic tumors called paranasal sinus mucoceles develop as a result of blocked sinus ostia. Mucus buildup promotes sinus bone wall extension and mass enlargement, which are thought to be prerequisites for this entity. Osteolysis is aided by prostaglandins and collagenases, which also make the cyst more expansile. They are typically benign and slow-growing, with the exception of pyeloceles, and have the potential to cause local morbidity on adjacent structures due to mass effect [9]. The majority of paranasal sinus mucoceles appear in the third or fourth decades of life, with a slight tendency toward the male sex [2,7]. Frontal and ethmoid paranasal sinuses are the most commonly affected sinuses [4].

The maxillary sinus and the sphenoid sinus are involved in a minority of cases [4]. Inflammation, trauma, or tumor distortion of sinus outflow pathways are the most common causes of cyst formation. Primary causes of ostia obstruction include cystic dilatation of mucosal glands and polyp degeneration, in addition to inflammatory obstruction. Secondary reasons are usually caused by prior sinus surgery or facial injuries. The etiology is not clarified, but the most commonly identifiable cause of mucoceles is FESS trauma [1,2], as shown in one patient in our study. Neoplasms, surgery, and allergies might lead to mucocele development [6,10].

In our study, mucocele had a higher incidence in males than in females (6:2), which is in contrast to the incidence reported by other studies [2,7]. Patients may be referred with symptoms ranging from mild to severe, depending on the location of the lesion, the extent of the bone defect, and symptoms caused by compression, such as pain and nasal congestion, eye edema, diplopia, loss of visual acuity, and intracranial problems. The clinical symptoms of mucocele vary and are not specific. The most common symptom reported by far is headache [2,7]. All patients in our study who had frontal and frontoethmoidal mucoceles initially complained of frontal headache and ophthalmic symptoms, as summarized in Table 1. Ophthalmologic symptoms were the most commonly identifiable symptoms in all patients. Impingement on adjacent orbital structures caused ophthalmic sequelae such as double vision, which was commonly followed by orbital swelling, epiphora, proptosis, and ptosis. The majority of the patients in our series had a history of previous sinus surgery, with no significant variations depending on the location of the mucocele.

According to Scangas et al., 44.9% of mucoceles had intraorbital or intracranial extensions, or both [2]. CT was performed preoperatively and postoperatively for all patients in our study because the method is important for obtaining information on the basic anatomy of the mucocele and for proper surgical planning. Postoperative CT can detect bone erosions, osteosclerotic changes, and calcification [1,6,7]. CT of the paranasal sinus mucocele shows an expansile, homogenous sinus mass that is not rim-enhancing [1]. When viewed radiographically, CT scans show the mucocele's basic anatomical details, outline how it interacts with nearby bony structures, and help with surgical planning. When an acute mucopylocele is present, a CT scan reveals an expansile, uniform sinus mass that does not enhance the rim. Although bone remodeling and growth are more frequently reported in connection with mucocele than bone degeneration [6].

Magnetic resonance imaging (MRI) is used to determine the mucocele’s duration and to distinguish it from neoplastic lesions that can obstruct natural sinus openings and impede drainage [6]. During early development, the mucocele content is typically aqueous, which results in hypointensity on T1-weighted MRI and hyperintensity [1]. The lesion is confirmed as benign respiratory mucosa with histopathological inspection of a slide stained with hematoxylin and eosin [1,8].

Mucoceles are usually surgically removed. The proper surgical method is determined by the location, magnitude, and expansion of the lesion. The endoscopic technique is a speedier operation with less morbidity, less damage to the nasal anatomy and physiology, and a shorter time before the patient can return to their daily routine. Definitive treatment options for paranasal sinus mucoceles include external approaches and endoscopic marsupialization [2]. There are certain contraindications for endoscopic surgery, such as severely altered postoperative anatomy that impede good visualization and extreme propagation of the mucocele to the intracranial space [7]. In this study, all patients underwent FESS without complications such as numbness, meningitis, projectile vomiting, cerebrospinal fluid (CSF) leak, third nerve affection, decrease, eye swelling, or hyposmia. Only headache was reported postoperatively for a short duration in five patients. In their study, Scangas et al. reported four postoperative complications [2]. One patient presented with protracted maxillofacial pain, the second patient presented with dacryocystitis and epiphora, the third patient presented with recurrent frontal sinus mucocele and osteomyelitis, and the fourth patient developed a small CSF leak while tracking the intact mucosa intraoperatively.

Post-operative treatment consists of irrigation of the nasal cavity with isotonic saline several times each day. A brief course of oral corticosteroids should be offered when paranasal sinus inflammation is identified intraoperatively. Clinical endoscopic examinations should be performed on a frequent and extended basis as part of the follow-up. A follow-up CT should be conducted if there is a clinical suspicion of recurrence.

One of the limitations of the study was that we did not perform CT scans for all the patients and mentioned only those which are available. A continuous follow-up period is considered an economic and preventive way to secure a good quality of life for patients suffering from paranasal mucoceles. The reported recurrence rate varies from one study to the other but is <10% of the reported cases [7,1]‏. The overall recurrence in our study was zero.

## Conclusions

The clinical symptoms of mucoceles vary, but the most common symptom is headache. The etiology is not clarified, but the most frequently identifiable cause of mucoceles is FESS. CT and MRI are the imaging methods that can help select the appropriate surgical approach. Endonasal endoscopic surgery is the definitive treatment option. The etiology of paranasal mucocele and the state of its prevalence in the Kingdom of Saudi Arabia should be investigated. Paranasal mucocele prevention, complication minimization, and its management are of interest and warrant further studies. If the mucoceles are big and there appears to be considerable bone erosion, producing orbital or intracranial problems, more aggressive procedures are required. Continuous follow-up should be considered to monitor the recurrence. Prevention and identification of recurrent mucocele are key obstacles in the therapy of this condition, which is why we advocate regular and prolonged clinical follow-up to discover lesions while they are still asymptomatic, before complications develop.
